# Auxin-induced *AUXIN RESPONSE FACTOR4* activates *APETALA1* and *FRUITFULL* to promote flowering in woodland strawberry

**DOI:** 10.1038/s41438-021-00550-x

**Published:** 2021-05-01

**Authors:** Xiangxiang Dong, Yanjun Li, Yuhan Guan, Shaoxi Wang, He Luo, Xiaoming Li, He Li, Zhihong Zhang

**Affiliations:** 1 Liaoning Key Laboratory of Strawberry Breeding and Cultivation, College of Horticulture, Shenyang Agricultural University, Shenyang, 110866 China; 2 Analytical and Testing Center, Shenyang Agricultural University, Shenyang, 110866 China

**Keywords:** RNAi, Gene regulation, Auxin

## Abstract

Flowering time is known to be regulated by numerous pathways, such as the autonomous, gibberellin, aging, photoperiod-mediated, and vernalization pathways. These regulatory mechanisms involve both environmental triggers and endogenous hormonal cues. Additional flowering control mechanisms mediated by other phytohormones, such as auxin, are less well understood. We found that in cultivated strawberry (*Fragaria* × *ananassa*), the expression of *auxin response factor4* (*FaARF4*) was higher in the flowering stage than in the vegetative stage. Overexpression of *FaARF4* in *Arabidopsis thaliana* and woodland strawberry (*Fragaria vesca*) resulted in transgenic plants flowering earlier than control plants. In addition, *FveARF4*-silenced strawberry plants showed delayed flowering compared to control plants, indicating that *FaARF4* and *FveARF4* function similarly in regulating flowering. Further studies showed that ARF4 can bind to the promoters of the floral meristem identity genes *APETALA1* (*AP1*) and *FRUITFULL* (*FUL*), inducing their expression and, consequently, flowering in woodland strawberry. Our studies reveal an auxin-mediated flowering pathway in strawberry involving the induction of *ARF4* expression.

## Introduction

Flowering marks a transition from vegetative to reproductive growth in plants and involves numerous physiological processes, metabolic pathways, and gene regulatory mechanisms^1,2^. These mechanisms involve intracellular and intercellular signal transduction cascades and the specific spatiotemporal expression of flowering genes^3–5^. To elucidate the molecular underpinnings of flowering, many studies have been performed on the model species *Arabidopsis thaliana*, resulting in the identification of genes involved in flowering regulation, as well as multiple signaling pathways: photoperiodic, vernalization, ambient temperature, autonomous, aging, and gibberellin (GA)^6–8^. However, it is recognized that given the interactions among phytohormones in regulating plant development, other signaling systems may also be involved^9^. For example, auxin is known to control many processes of plant growth and development and broadly regulates gene expression; however, its role in flowering and the associated molecular mechanisms remain poorly understood^10,11^.

Central to auxin-regulated transcription are three families of primary auxin-responsive genes: Aux/IAA (AUXIN/INDOLE ACETIC ACID), GH3 (GRETCHEN HAGEN3), and SAUR (SMALL AUXIN UP RNA)^12^. All of the gene promoters from these families contain the TGTCNC motif (auxin response element, AuxRE), which is bound to AUXIN RESPONSE FACTOR (ARF) proteins to mediate auxin responses^13^. In *A. thaliana*, ARF functions have been well studied, and it is known that *AtARF1*, *AtARF2*, *AtARF6*, and *AtARF8* are involved in floral organ development^14^, while *AtARF7*, *AtARF16*, and *AtARF19* are associated with root development^13,15^, *AtARF12*-*15* regulates embryogenesis and seed development, and *AtARF20*-*22* has similar functions to *AtARF12*-*15*^13,16^.

*AtARF3* and *AtARF4* are involved in the transition from vegetative to reproductive growth, and a unique feature of *ARF3*/*4* regulation is that their transcripts are post-transcriptionally cleaved by *trans*-acting short-interfering RNAs, such as *tasiRNA3*, an endogenous *trans*-acting small-interfering RNA^13^. The production of *tasiRNA3* is initiated by cleavage of the non-protein-coding *TAS3* RNA by miR390. A complex of the miR390-cleaved transcript bound to ARGONAUTE7 (AGO7) is then used as a template for polymerization by RNA-DEPENDENT RNA POLYMERASE6 (RDR6) and SUPPRESSOR OF GENE SILENCING3 (SGS3)^10^. The resulting double-stranded RNA is cleaved by DICER-LIKE4 (DCL4) to generate the 21-nucleotide-long *tasiRNA3*^17^. Thus, AGO7/RDR6/SGS3/DCL4 is required for the production and/or stability of *tasiRNA3*, which targets both *ARF3* and *ARF4*. Mutations in *AGO7*/*RDR6*/*SGS3*/*DCL4* in *A. thaliana* accelerate the juvenile-to-adult transition, and the expression of *ARF3* and *ARF4* is upregulated in the *ago7*/*rdr6*/*sgs3*/*dcl4* mutant^17^. This indirectly demonstrates that the *ago7*/*rdr6*/*sgs3*/*dcl4* phenotype is attributable to the absence of *tasiRNA3*-mediated repression of *ARF3* and *ARF4*^10^. However, the specific function of *AtARF3* and *AtARF4* in the phase transition is unclear.

A potentially useful experimental system to study flowering transition is strawberry (*Fragaria*)^18^. Early flowering is a great advantage for fruit crop breeding and cultivation since it shortens the process of generating new cultivars and reduces the time to produce a marketable crop, resulting in economic benefits^7,19^. Octoploid cultivated strawberry (*Fragaria* × *ananassa*) is widely cultivated in all arable regions around the globe and has high economic value^20^. However, although *F.* × *ananassa* is of great agricultural and research importance, genetic transformation and associated functional studies are hindered by its complex genetics, which include a large genome and high degree of heterozygosity^21^. In contrast, diploid woodland strawberry (*Fragaria vesca*) is suitable for regeneration, and a transformation system has been established in vitro^22^. Moreover, *F. vesca* has a substantial seed set, rapid growth, a small size, a relatively small genome, and existing genetic maps, which makes it a good option as a model plant for the development of genomic tools, enabling us to perform functional studies using forward or reverse genetic approaches^23^. Notably, *F. vesca* shares considerable sequence similarity with *F.* ×*ananassa* and other rosaceous plants, and the high levels of functional conservation at the genetic level mean that knowledge of diploid strawberry gene function has the potential to be applied to octoploid strawberry or other members of the Rosaceae family^7,23^.

In this study, we investigated the role and mechanism of auxin-regulated flowering in strawberry and found that the expression level of auxin-induced *FaARF4* was upregulated in the flowering stage rather than during vegetative growth, indicating that *FaARF4* is related to flowering. Using transgenic plants, we discovered that *FaARF4* promoted flowering and that *FveARF4*-silenced plants showed delayed flowering. Further studies revealed that FaARF4 and FveARF4 bound to the promoters of *FveAP1* and *FveFUL* to induce their expression. Our findings showed that auxin is involved in the flowering pathway in strawberry through its regulation of *ARF4* expression.

## Results

### *FaARF4* expression analysis

**Fig. 1:**
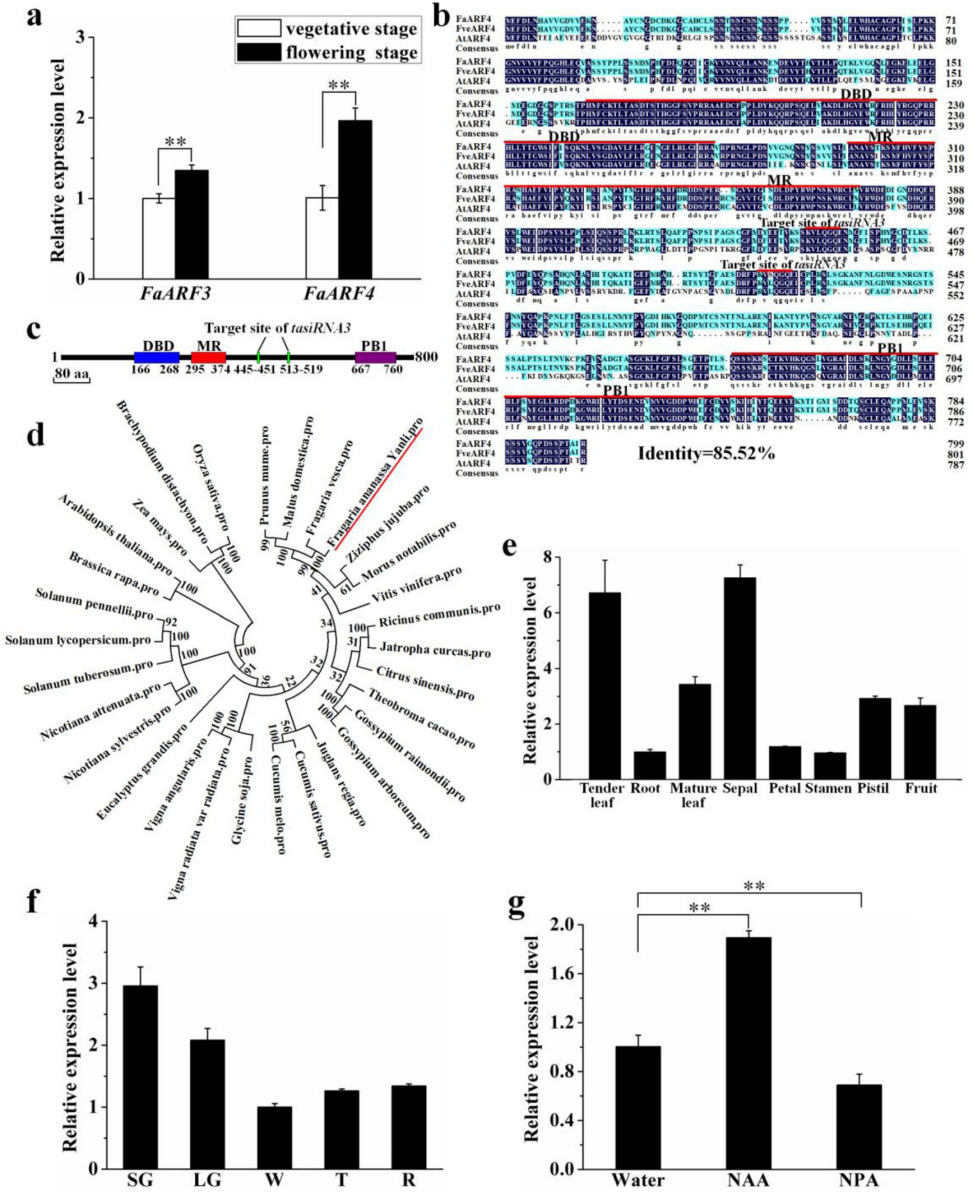
Expression pattern, sequence alignment, and phylogenetic analysis of the *FaARF4* gene or cognate protein. **a** The expression levels of *FaARF3* and *FaARF4* during the flowering and vegetative stages. **b** Amino acid sequence alignment of FaARF4, FvARF4, and AtARF4. **c** DNA binding domain (DBD), middle region (MR), and protein binding (PB1) domain structure in FaARF4. **d** Phylogenetic tree of FaARF4 with other ARF4 proteins from different plant species. Expression of *FaARF4* in different tissues (**e**), different stages of fruit development (**f**), and ‘Yanli’ strawberry treated with α-naphthalene acetic acid (NAA) (**g**). Vertical bars represent the SDs (*n* = 3). ^**^p < 0.01

Elite strawberry cultivars, free of plant pathogenic fungi and bacteria, are commonly produced by micropropagation, although several problems, such as epigenetic variation, can be encountered using this approach^24^. In our previous study, we reported that the flowering characteristics of micropropagated strawberry (*F*. × *ananassa*) changed so that it exhibited early flowering, along with a low level of miR390 expression^25^. Next, we overexpressed miR390 in tobacco and found that the juvenile-to-adult phase transition was significantly delayed^26^. Since miR390 negatively regulates the expression of *ARF3*/*4*, we speculated that *ARF3*/*4* may be related to flowering in cultivated strawberry. To study the expression patterns of *FaARF3* and *FaARF4* in response to flowering, we determined their expression profiles in stem tips at different developmental stages in the cultivated strawberry ‘Yanli’ by quantitative reverse transcription-polymerase chain reaction (qRT-PCR) analysis. As shown in Fig. 1a, the expression levels of *FaARF3* and *FaARF4* were higher in the flowering stage than in the vegetative stage, indicating that these two genes are involved in flowering. Since the expression level of *FaARF4* changed more than that of *FaARF3*, *FaARF4* was chosen for further study.

### Cloning and phylogenetic analysis of *FaARF4*

Primers for *FaARF4* from the cultivated strawberry ‘Yanli’ were designed based on the *FveARF4* sequence in the NCBI database (https://www.ncbi.nlm.nih.gov/). The full-length coding sequence (CDS) of *FaARF4* (GenBank No. MG765454) was found to be 2403 bp long and encode an 800-amino-acid protein with many α-helices, β-sheets, and hydrophilic and flexible regions (Fig. S1). An alignment of the amino acid sequences of FaARF4, FveARF4, and AtARF4 revealed 85.52% sequence identity (Fig. 1b). The conserved regions were primarily confined to three domains, the DNA binding domain (DBD), the middle region (MR), and the protein binding (PB1) domain, consistent with FaARF4 being a member of the ARF family. As shown in Fig. 1c, FaARF4 contains a DBD, an MR, a PB1, and two *tasiRNA3* target sites.

To study the phylogenetic relationships of ARF4 with different species, the ARF4 amino acid sequences from members of the Rosaceae and other plant species were retrieved from the NCBI database. A phylogenetic analysis (Fig. 1d) showed that the FaARF4 and FveARF4 sequences were the most closely related, followed by other Rosaceae proteins and then proteins from other species, consistent with their phylogenetic placement.

### *FaARF4* expression profile in strawberry

qRT-PCR analysis of *FaARF4* in different strawberry tissues revealed that this gene was universally expressed; the highest expression level was found in sepals, where the expression level was approximately 7-fold higher than that in roots, which had the lowest expression level (Fig. 1e). Next, we measured *FaARF4* expression during fruit development and ripening. Strawberry fruit maturity can be divided into five stages: small green (SG), large green (LG), white (W), turning (T), and red (R)^27^. As shown in Fig. 1f, *FaARF4* was strongly expressed in the SG stage, followed by a gradual decline until the W stage, with no change in the T and R stages. *FaARF4* expression in the SG stage was ~3-fold higher than that in the W stage.

To test the hypothesis that auxin controls *FaARF4* expression, we analyzed its expression in ‘Yanli’ fruit after treatment with water (control), 500 μM NAA (α-naphthalene acetic acid, a synthetic auxin), or 500 μM NPA (N-1-naphthylphthalamic acid, an auxin inhibitor) by qRT-PCR. As shown in Fig. 1g, the expression of *FaARF4* was upregulated after NAA treatment and downregulated after NPA treatment, suggesting that auxin induces the expression of *FaARF4*.

### Identification of the transcription factor *FaARF4*

**Fig. 2:**
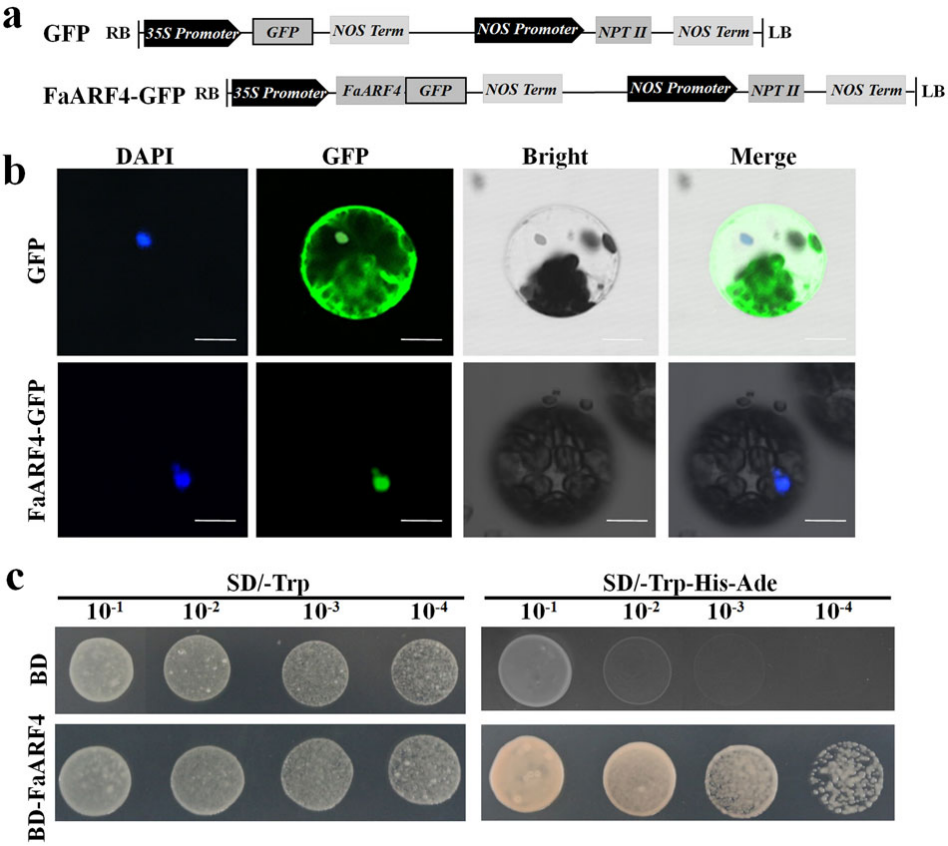
FaARF4 transcription factor characteristics. **a** Structural diagram of the pRI 101-AN-GFP vector and the pRI 101-AN-FaARF4-GFP construct. **b** Subcellular localization of FaARF4 in *Arabidopsis thaliana* protoplasts. *A. thaliana* cell nuclei were identified using 4ʹ,6-diamidino-2-phenylindole (DAPI) staining. Scale bars = 25 μm. **c** FaARF4 transcriptional activity

FaARF4 sequence analysis predicted that it localizes to the nucleus (http://www.csbio.sjtu.edu.cn/bioinf/Cell-PLoc/), and this hypothesis was tested by expressing a green fluorescent protein (GFP):ARF4 fusion (FaARF4-GFP) driven by the constitutive CaMV 35S promoter (Fig. 2a) in *A. thaliana* protoplasts and *Nicotiana benthamiana* leaves. A constitutively expressed GFP control was similarly tested. The green fluorescent signal of FaARF4-GFP was only detected in the nucleus, while GFP alone was found throughout the entire cell (Figs. 2b and S2), suggesting that FaARF4 specifically accumulates in the nucleus.

To investigate whether FaARF4 has transcriptional activation activity, we fused its CDS with the GAL4 DBD and created the pGBT9-FaARF4 construct (BD-FaARF4). BD-FaARF4 and the empty vector pGBT9 (BD), as a negative control, were then transformed into yeast for transcriptional activity analysis. As shown in Fig. 2c, the yeast strain containing BD-FaARF4 grew well on SD/-Trp-His-Ade media, while the yeast strain containing BD grew only in SD/-Trp, demonstrating that FaARF4 is a transcriptional activator.

### Mutation of *FaARF4 tasiRNA3* target sites

**Fig. 3:**
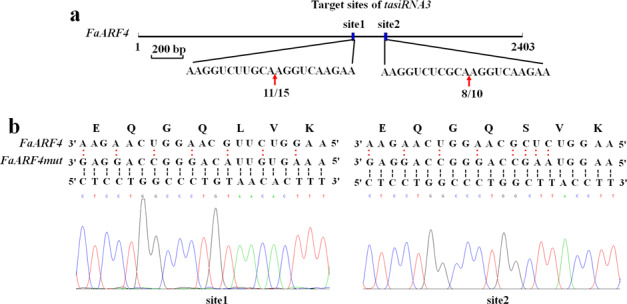
Rapid PCR site-directed mutagenesis of *FaARF4*. **a** RLM-5ʹ RACE assay showing that *tasiRNA3* cleaves *FaARF4*. The red arrows represent the cleavage sites. The numbers under the red arrows represent the number of clones that possessed the cleavage site and the total number of clones in the RLM-5ʹ RACE assay. **b** Nucleotide and amino acid sequence analysis of *FaARF4mut* and *FaARF4*. The red colons indicate the nucleotides that differ between *FaARF4mut* and *FaARF4*. The dotted lines represent complementary sequences

*tasiRNAs* are a group of endogenous non-coding small RNAs that function on their target genes by complementary base pairing. It is well known that *ARF4*, the target gene of *tasiRNA3*, is negatively regulated by *tasiRNA3* in *A. thaliana*^10,13^. To verify whether *tasiRNA3* cleaves *FaARF4* in strawberry, an RLM-5ʹ rapid RACE assay was performed. As shown in Fig. 3a, *FaARF4* contains two *tasiRNA3* target sites (site 1 and site 2) located in the coding region that were cleaved by *tasiRNA3*. To prevent *FaARF4* cleavage by endogenous *tasiRNA3*, we performed rapid PCR site-directed mutagenesis of *FaARF4* using the methods of Picard et al. (1994)^28^. We obtained the nucleotide sequence of a *tasiRNA3*-insensitive mutant (named *FaARF4mut*) and confirmed that the amino acid sequence was the same as that of FaARF4 and that only the nucleotide sequence was affected (Fig. 3b). *FaARF4mut* was used for further functional studies.

### *FaARF4* promotes flowering in *A. thaliana* and woodland strawberry

**Fig. 4:**
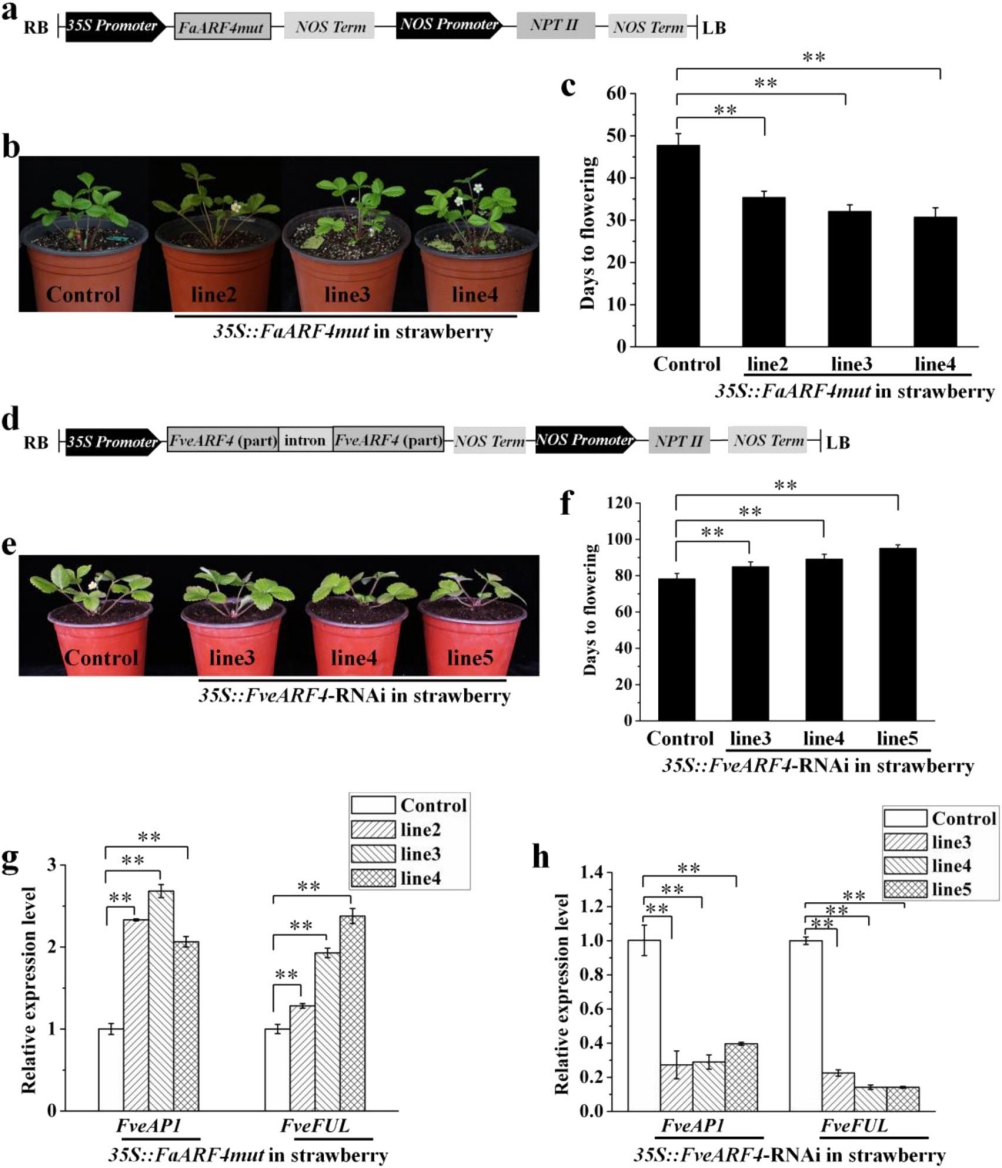
Phenotypic observation and flowering time analysis of transgenic strawberry plants. **a** Structural diagram of the vector used to overexpress *FaARF4* driven by the CaMV 35S promoter. *FaARF4*-overexpressing plants showed early flowering (**b**), and the number of days until flowering was less than that for the control plants (**c**). Vertical bars represent the SDs of approximately 10 plants (^**^p < 0.01). **d** Structural diagram of the *FveARF4* RNAi vector driven by the CaMV 35S promoter. By observing *FveARF4* RNAi plants (**e**), we found that the number of days until flowering was greater than that of the control plants (**f**). Vertical bars represent the SDs of approximately 10 plants (^**^p < 0.01). The expression of *FveAP1* and *FveFUL*, which are tissue-specific genes expressed in the flower meristem, increased in *FaARF4*-overexpressing plants (**g**) and was reduced in *FveARF4* RNAi plants (**h**). Vertical bars represent the SDs (*n* = 3). ^**^p < 0.01

*A. thaliana* is often used to assess the function of genes from other plant species^20^, and here we transformed both *A. thaliana* (Columbia) and ‘Ruegen’ (*F. vesca*) with the pRI 101-*FaARF4mut* plasmid (Fig. 4a). All transgenic lines were identified by detection of the *FaARF4* gene using the 35S and ARF4-R primers. A 2000–3000 bp band was amplified from the genomic DNA of all transgenic lines, and no corresponding bands were amplified from control plants (Fig. S3a). We obtained four transgenic *A. thaliana* lines and four transgenic strawberry lines. *FaARF4* expression was investigated in the transgenic lines and control plants, and it was clearly higher in the transgenic plants (Fig. S3b, c).

The phenotypic differences between the transgenic lines and wild-type *A. thaliana* plants are shown in Fig. S3d. We found that transgenic plants flowered earlier than the wild-type and that the vegetative growth stage was approximately 52 days for the wild-type and 44 days for the transgenic plants (Fig. S3e). These results indicated that *FaARF4* can regulate the flowering process in *A. thaliana*.

To better observe the phenotype of the transgenic strawberry plants, we transplanted them into a greenhouse. We observed an early flowering phenotype in the transgenic lines (Fig. 4b) and found that the transgenic plants flowered 15 days earlier than the control plants (Fig. 4c). Thus, *FaARF4* also regulates flowering in strawberry.

### *FveARF4* silencing inhibits flowering in woodland strawberry

FaARF4 and FveARF4 have a high degree of sequence identity (99.47%), suggesting similar functions (Fig. 1b, d). To confirm the function of *FveARF4*, an RNAi vector was created and transformed into ‘Ruegen’ using *Agrobacterium*-mediated transformation (Fig. 4d). Transgenic lines were confirmed by PCR amplification of a 250–500 bp band that was not amplified from control plants (Fig. S3f). We also analyzed *FveARF4* expression by qRT-PCR in the RNAi lines. Compared with the control plants, the expression of *FveARF4* in the RNAi lines was lower (Fig. S3g). We observed that the flowering time of the RNAi lines was delayed by 8 days compared to that of the control plants, indicating that *FveARF4* regulates the flowering process in strawberry (Fig. 4e, f).

### *ARF4* directly binds to the promoters of *FveAP1* and *FveFUL* in strawberry

In *A. thaliana*, the floral integration genes *FLOWERING LOCUS T* (*FT*) and *SUPPRESSOR OF OVEREXPRESSION OF CONSTANS1* (*SOC1*) initiate flowering by activating tissue-specific flower meristem genes, including *LEAFY* (*LFY*), *APETALA1* (*AP1*), and *FRUITFULL* (*FUL*); in addition, *TERMINAL FLOWER1* (*TFL1*) inhibits the expression of *LFY* and *AP1*, and plays an important role in regulating the flowering process^1,7^. To explore the cause of early flowering in strawberry, we measured the expression of these flowering-related genes. qRT-PCR analysis showed that the expression levels of *FveAP1* and *FveFUL* were higher in *FaARF4*-overexpressing plants than in control plants (which was set to 1) (Fig. 4g), while the expression of *FveFT*, *FveSOC1*, *FveLFY*, and *FveTFL1* was not significantly different between transgenic plants and control plants (Fig. S4). *FveAP1* and *FveFUL* expression was lower in the RNAi lines than in control plants (Fig. 4h).

**Fig. 5:**
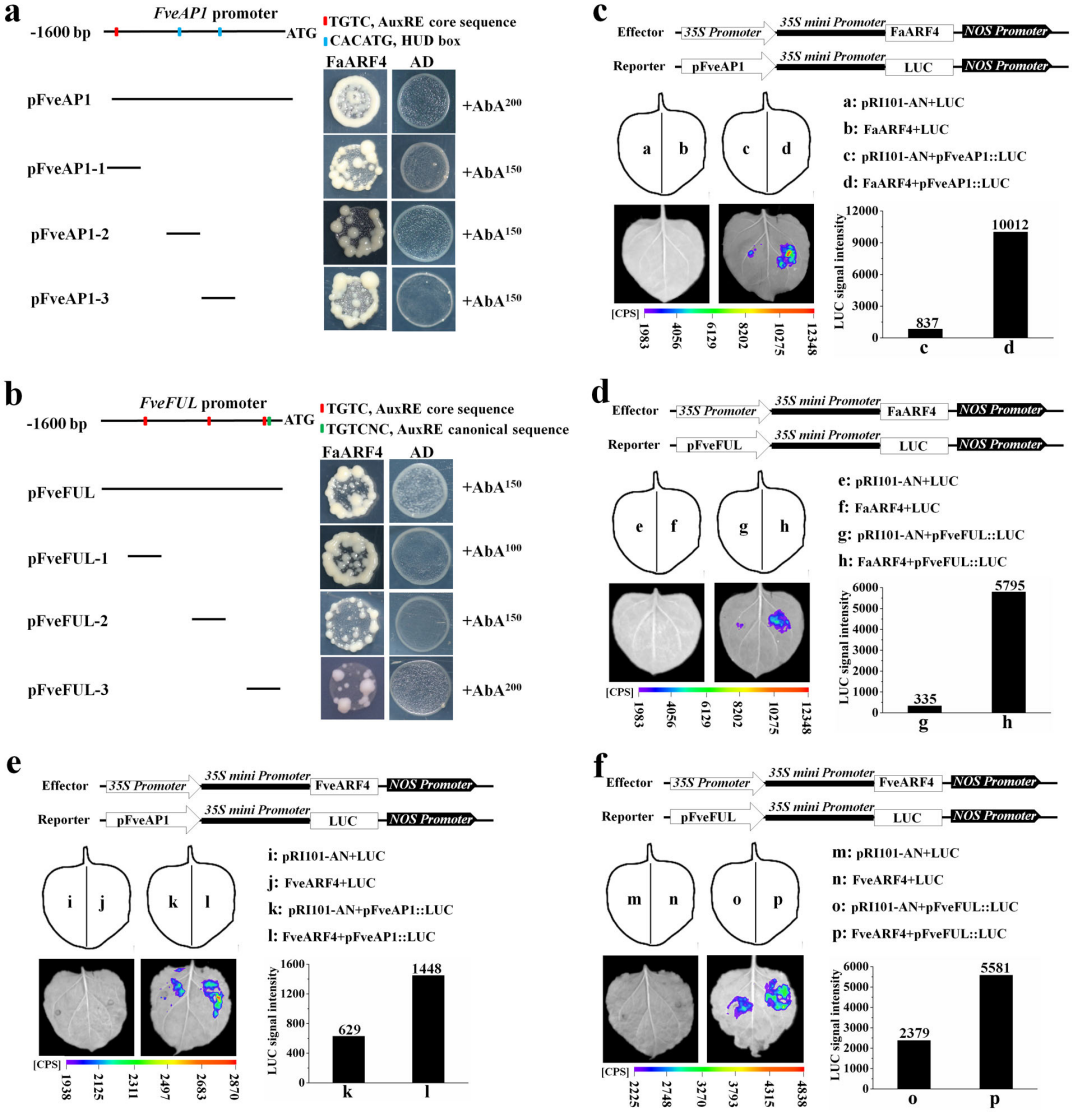
ARF4 positively regulates *FveAP1* and *FveFUL* expression by binding to their promoters. Yeast one-hybrid (Y1H) assay showing that *FaARF4* binds to *FveAP1* promoter fragments (pFveAP1, pFveAP1-1, pFveAP1-2, and pFveAP1-3) (**a**) and *FveFUL* promoter fragments (pFveFUL, pFveFUL-1, pFveFUL-2, and pFveFUL-3) (**b**) in vitro. The empty pGAD424 and pAbAi vectors were used as negative controls. In vivo luciferase reporter assay showing that FaARF4 and FveARF4 positively regulate *FveAP1* expression (**c**, **e**) and *FveFUL* expression (**d**, **f**), respectively. The FaARF4/FveARF4 effector and either the pFveAP1 or the pFveFUL reporter were coinfiltrated into tobacco leaves, and the luciferase signal was measured

Previous studies have shown that ARF proteins can also bind to G boxes (CACGTG) and HUD boxes (CACATG) in addition to the AuxRE^29^. As shown in Fig. 5a, the *FveAP1* promoter is predicted to have one AuxRE and two HUD boxes distributed across four regions (pFveAP1, pFveAP1-1, pFveAP1-2, and pFveAP1-3). In addition, the *FveFUL* promoter contains four AuxREs distributed across four regions (pFveFUL, pFveFUL-1, pFveFUL-2, and pFveFUL-3), as shown in Fig. 5b.

To verify whether FaARF4 binds to the *FveAP1* and *FveFUL* promoters, yeast one-hybrid (Y1H) analysis and luciferase reporter assay were carried out. The Y1H assay indicated that FaARF4 bound to the AuxRE and HUD boxes in the *FveAP1* and *FveFUL* promoters in vitro (Fig. 5a, b). The *FveAP1* and *FveFUL* promoters were then individually inserted into the pRI-mini35S-LUC vector (luciferase reporter vector) as reporters, and FaARF4 driven by the 35S promoter was used as the effector (Fig. 5c, d) in a transient tobacco leaf expression assay. We observed that FaARF4 also bound to the *FveAP1* and *FveFUL* promoters in vivo (Fig. 5c, d). The Y1H and luciferase reporter assays suggested that FaARF4 directly binds to the *FveAP1* and *FveFUL* promoters, and activates the transcription of the corresponding genes. Since the expression of *FveAP1* and *FveFUL* was reduced in the RNAi lines, we speculated that FveARF4 and FaARF4 had the same mechanism. Supporting this hypothesis were the results of the transactivation activity assay demonstrating that FveARF4 is also a transcriptional activator (Fig. S5) and those of the luciferase reporter assay indicating that FveARF4 can bind to the promoters of both *FveAP1* and *FveFUL* (Fig. 5e, f).

## Discussion

### *ARF4* is involved in the IAA-mediated flowering pathway in strawberry

In species such as sweet cherry (*Prunus avium*)^30^ and longan (*Dimocarpus longan*)^7^, shortening the life cycle can allow multiple generations to be grown commercially in a single season, and the breeding process can be accelerated. It is also possible to increase the yield of plants by prolonging the number of days of vegetative growth in species such as sugar beet (*Beta vulgaris*)^4^ and perennial sugarcane (*Saccharum officinarum*)^3^. Moreover, flowering genes can also be used to adjust the flowering period, which greatly improves the economic value of ornamental plants^31^. In cultivated strawberry, the regulation of flowering is used to provide fresh berries throughout the year^32^. Most strawberry cultivars are June-bearing, and their flower formation is affected by environmental conditions, especially light and temperature^18^. Growth studies have revealed that short days and/or cool temperatures can promote flowering, and short days greatly promote and enhance flower initiation^19,33^. At 15 °C or during crown-cooling treatments, flowering time was hastened, and the number of flowers increased^34^. It was also reported that light quality affected flowering time and that strawberry could be induced to flower when exposed to far red light^33^. The role of GA in strawberry flowering has also been studied, and it has been shown to inhibit flowering and promote the formation of runners^32^. Additionally, it was reported that both auxin IAA and cytokinin affect the type of first bud initiation, and exogenously applied IBA (indole-3-butytric acid, an IAA analog) +6-BA (6-benzylaminopurine, a synthetic cytokinin) leads to the production of inflorescences instead of runners^35^. However, it has not been reported how IAA regulates flowering at the molecular level.

IAA regulates plant growth and development by employing signal transduction, and as the center of the auxin signaling pathway, the expression of ARF proteins is known to be regulated by IAA^13^. The expression of *ARF4* is also affected by IAA, and it has been shown to be upregulated by IAA treatment in model plants, such as *A. thaliana*^36^, *Medicago truncatula*^37^, and *Brachypodium distachyon*^38^. In a previous study, we also demonstrated that the expression of *FveARF4* was induced by IAA in woodland strawberry^39^, and here, we found that the expression of *FaARF4* was increased by IAA and reduced by NPA (Fig. 1g). We analyzed the function of both *FaARF4* and *FveARF4* and found them to be involved in the regulation of flowering time, which helps to elucidate the IAA-mediated flowering pathway in strawberry (Fig. 4a–f).

### *ARF4* promotes flowering by directly activating the expression of *FveAP1* and *FveFUL* in strawberry

**Fig. 6:**
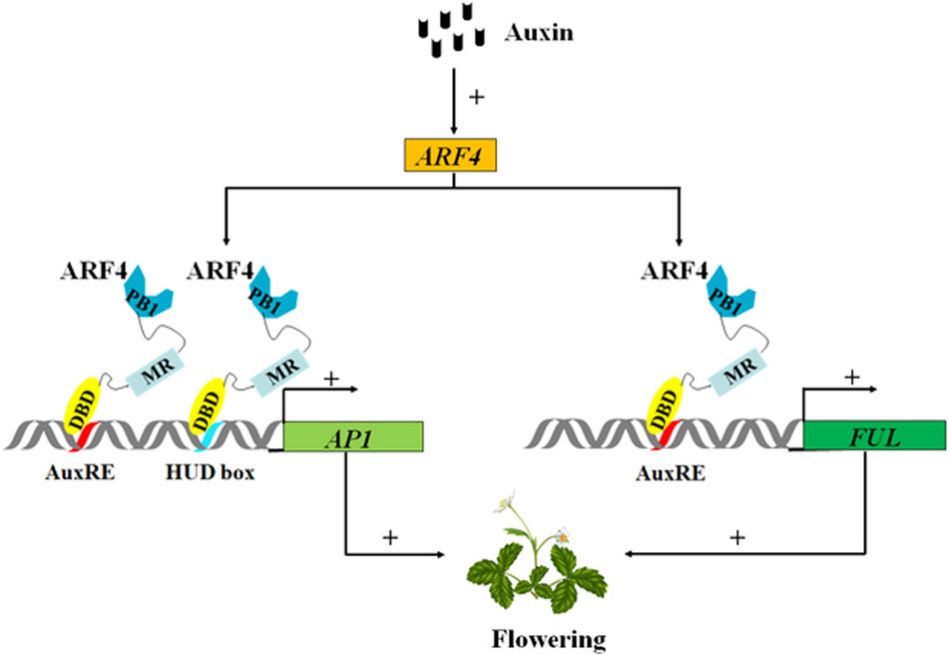
Model of how auxin induces *ARF4* to promote the flowering pathway. In woodland strawberry, auxin induces the expression of *ARF4*, and the ARF4 protein binds to and activates the *AP1* and *FUL* promoters. The expression of *AP1* and *FUL* initiates flowering. Symbols/abbreviations: ‘+’, promotion; AuxRE and HUD box, auxin-responsive element (ARF4 binding sites)

In *A. thaliana*, flowering pathways involving endogenous cues, exogenous cues, and intrinsic genetic programs govern flowering time^7^. Based on the analysis of loss-of-function mutants and transgenic plants, approximately 180 genes have been shown to be involved in the regulation of flowering time, including *AtARF3* and *AtARF4*^10,13^. It has been suggested that *AtARF3* and *AtARF4* indirectly activate *AtLFY*, *AtAP1*, and *AtFUL* by enhancing the expression of *AtSPL3*, the target gene of miR156, which promotes flowering in *A. thaliana*, but this has not been experimentally confirmed^6^. However, we observed no *FveSPL3* transcriptional activation activity in yeast (Li, 2020; unpublished data). To explore how *ARF4* regulates flowering in woodland strawberry, we analyzed the expression of flowering-related genes in transgenic and control strawberry and observed significant differences in the expression levels of *FveFUL* and *FveAP1* (Fig. 4g, h). We also showed that FaARF4 and FveARF4 have transcriptional activation activity (Figs. 2b and S5), and Y1H and luciferase reporter assays further confirmed that ARF4 can bind to the AuxRE and HUD-box motifs in the *FveFUL* and *FveAP1* promoters and activate their expression (Fig. 5). *FUL* and *AP1* belong to the *AP1*/*SQUA* gene subfamily and are key genes in flowering regulation, and it has been reported that they promote flowering in various species, such as *A. thaliana*^7^, soybean (*Glycine max*)^40^, tomato (*Solanum lycopersicum*)^41^, maize (*Zea mays*)^42^, apple (*Malus domestica*)^43^, and others. Notably, the expression of *FUL* and *AP1* is important in controlling flowering in woodland strawberry: high expression of *FUL* and *AP1* promotes flowering, and if they are not highly expressed, vegetative growth continues^18^. In addition, we found that flowering time correlated with the expression levels of *FveFUL* and *FveAP1* in transgenic strawberry lines (Fig. 4g, h). Taken together, we propose an IAA-mediated flowering pathway in strawberry (Fig. 6), which suggests a target for altering flowering time for horticultural purposes.

Flowering is an important factor in the production of fruit trees, and the existence of a long juvenile phase in fruit trees has been suggested to limit breeding and genetic improvement^32^. Furthermore, most economically important phenotypes related to fruits of hybrid cultivars cannot be identified in this phase, and one of the major goals of fruit breeding programs is to reduce the juvenile phase and accelerate floral production^18^. Genetic engineering has been used to successfully shorten the juvenile phase and promote flowering in several fruit species^7^. In our study, we found that the auxin-induced *FaARF4* and *FveARF4* genes were involved in the regulation of flowering. ARF4 has a sequence similar to those of homologs from fruit tree species, especially members of the Rosaceae, indicating analogous functions. Therefore, it may be possible to break the long juvenile phase of fruit trees using overexpression of the *ARF4* gene.

## Materials and methods

### Plant materials and treatments

The cultivated strawberry (*F.* × *ananassa*) cultivar ‘Yanli’ was grown under greenhouse conditions at Shenyang Agricultural University, China. Stem tips from the 4-leaf and bud stages were collected as vegetative stage and flowering stage samples, respectively. Different organs and fruits at different developmental stages were harvested individually for gene expression analysis, frozen in liquid nitrogen, and stored at –80 °C. In the white ripening stage, the fruits were picked and transported to the laboratory, where they were divided into three groups: fruits from one group were submerged in water (as the control); fruits from the second group were submerged in 500 μM NAA (Solarbio, Beijing, China); and fruits from the third group were submerged in 500 μM NPA (a polar auxin transport inhibitor; Shanghai Yuanye Bio-Technology Company Limited, Shanghai, China). All treatments lasted for 30 s. After 1 h, total RNA samples were extracted using the cetyltrimethyl ammonium bromide (CTAB)-based method for the analysis of *FaARF4* expression^44^. Three biological replicates were prepared.

The strawberry leaf transformation experiment was performed using ‘Ruegen’ (*F. vesca*), which was grown in tissue culture. *Agrobacterium*-mediated leaf transformation was carried out after plants had grown 4–5 leaves^45^.

Seeds of wild-type *Arabidopsis thaliana* Columbia were sown on peat:vermiculite:perlite::3:3:1 substrate after 3 days of vernalization at 4 °C and were placed in climate-controlled boxes. Transformation was carried out using the floral dip method^20^.

### Gene isolation

cDNA samples were synthesized from the total RNA described above using the RNA PCR Kit (Takara, Dalian, China) following the manufacturer’s instructions. The full-length *FaARF4* CDS was cloned by RT-PCR using the FaARF4-F and FaARF4-R primers (Table S1) with *Nde* I and *Sal* I restriction sites added at the 5ʹ and 3ʹ ends, respectively.

PCR was performed under the following conditions: 95 °C for 5 min, followed by 35 cycles of 95 °C for 30 s, 57 °C for 30 s, and 72 °C for 3 min, with a final extension at 72 °C for 5 min. The PCR products were ligated into the pMD18-T vector (Takara, Dalian, China) and subsequently transformed into *Escherichia coli* strain DH5α. Subsequently, positive colonies were selected, the gene sequence was amplified by PCR, and the product was sequenced at BGI-Shenzhen, China. The ExPasy ProtParam tool (http://web.expasy.org/compute_pi/) and DNASTAR program (DNASTAR Inc., USA) were used to predict the structure of the encoded proteins. The amino acid sequences of FaARF4, FveARF4, and AtARF4 were aligned using DNAMAN 6.0 software (Lynnon Biosoft, USA).

### Phylogenetic analysis

The ARF4 amino acid sequences from *A. thaliana* (NP_200853), *B. distachyon* (XP_003564986), *Brassica rapa* (XP_009120578), *Citrus sinensis* (XP_006488135), *Cucumis melo* (XP_008463923), *Cucumis sativus* (XP_030503928), *Eucalyptus grandis* (XP_010043718), *F. vesca* (XP_004309870), *Glycine soja* (XP_028190393), *Gossypium arboretum* (XP_017611878), *Gossypium raimondii* (XP_012443345), *Jatropha curcas* (XP_012064855), *Juglans regia* (XP_018819532), *M. domestica* (XP_008385290), *Morus notabilis* (XP_010104118), *Nicotiana attenuata* (XP_019229596), *Nicotiana sylvestris* (XP_009779766), *Oryza sativa* (XP_015650953), *Prunus mume* (XP_008225336), *Ricinus communis* (XP_015579153), *S. lycopersicum* (NP_001233771), *Solanum pennellii* (XP_015058398), *Solanum tuberosum* (XP_006340145), *Theobroma cacao* (XP_007015441), *Vigna angularis* (XP_017425709), *Vigna radiata* var. *radiata* (XP_014498033), *Vitis vinifera* (XP_002285019), *Z. mays* (XP_008656904), and *Ziziphus jujuba* (XP_024928576) were obtained from the NCBI nucleotide database (http://www.ncbi.nlm.nih.gov/nucleotide/). A phylogenetic tree was constructed using the neighbor-joining method using MEGA 6.0 software with 1000 bootstrap replicates to evaluate the reliability of the phylogenetic grouping. The tree files were viewed and edited using MEGA 6.0^46^.

### Quantitative RT-PCR analysis

Quantitative RT-PCR (qRT-PCR) was performed using the 7500 system (Applied Biosystems, Foster City, USA) according to the manufacturer’s instructions with SYBR Green II (Takara, Dalian, China) and the *FaARF3* and *FaARF4* qRT-PCR primers listed in Table S1 (FaARF3-QF/R and FaARF4-QF/R). The experiments were conducted on three biological replicates, and the results were normalized using strawberry 26S rRNA as the housekeeping gene^45^. The qRT-PCR analysis of *Fa26S* was conducted with the following primers: Fa26S-F and Fa26S-R (Table S1). Tender leaves from transgenic strawberry plants and transgenic *A. thaliana* plants were harvested for gene expression analysis. *Fve26S* was used as the housekeeping gene for woodland strawberry, and *AtACTIN8* was selected as the housekeeping gene for *A. thaliana*^20^ (Table S1).

The nucleic acid sequences of flowering-related genes were obtained from the Genome Database for Rosaceae (GDR) (https://www.rosaceae.org/species/fragaria/all). The qRT-PCR analysis of *FveSOC1* (FvH4_7g127000), *FveFT* (FvH4_6g00090), *FveLFY* (FvH4_5g09660), *FveAP1* (FvH4_4g29600), *FveFUL* (FvH4_5g13500), and *FveTFL1* (FvH4_6g18480) was conducted using six pairs of primers: FveSOC1-QF/R, FveFT-QF/R, FveLFY-QF/R, FveAP1-QF/R, FveFUL-QF/R, and FveTFL1-QF/R (Table S1). Stem tips of transgenic strawberry plants were used for gene expression analysis.

PCR was performed using the following thermal cycling conditions: 95 °C for 10 min, followed by 40 cycles of 95 °C for 10 s and 60 °C for 1 min. The transcription levels were calculated using the 2^–ΔΔCT^ method^47^.

### Subcellular localization

The full-length *FaARF4* CDS without the termination codon was amplified using LA Taq polymerase (Takara, Dalian, China) with gene-specific primers FaARF4-GFP-F and FaARF4-GFP-R (Table S1) harboring *Xba* I and *Xho* I sites. PCR was performed under the following conditions: 95 °C for 5 min, followed by 35 cycles of 95 °C for 30 s, 55 °C for 30 s, and 72 °C for 3 min, with a final extension at 72 °C for 5 min. The PCR products were inserted into the pGPTVII-GFP vector between the *Xba* I and *Xho* I sites to create the *35S*::*FaARF4*-*GFP* (FaARF4-GFP) construct. Using pGPTVII-GFP containing *35S*::*GFP* (GFP) as a control, vectors were injected into *A. thaliana* protoplasts and *N. benthamiana* leaves^45^. Two days after agroinfiltration, GFP fluorescence was imaged using a laser confocal fluorescence microscope (TCS SP8-SE; Leica, Wetzlar, Germany) with an excitation wavelength of 488 nm and a 505–530 nm bandpass emission filter.

### Transcriptional activation analysis

The *FaARF4* and *FveARF4* CDSs were individually inserted into the pGBT9 vector to create the pGBT9-FaARF4 (BD-FaARF4) and pGBT9-FveARF4 (BD-FveARF4) constructs, respectively. The primers used are listed in Table S1. BD-FaARF4, BD-FveARF4, and the empty vector pGBT9 (BD) were then transformed into yeast (*Saccharomyces cerevisiae*) for transcriptional activity analysis using the Yeast Transformation Kit (Shaanxi Protein Interaction Biotechnology Company Limited, Shaanxi, China). The transformed yeast strains were grown on SD/−Trp medium and selected on SD/−Trp–His–Ade medium. The transcriptional activity of FaARF4 was determined by observing yeast growth according to previously described methods^48^. X-α-gal (5-bromo-4-chloro-3-indolyl-α-D-galactoside) was added to the SD/−Trp–His–Ade medium, and the resulting blue coloration was used as an indication of FveARF4 transcriptional activation activity^49^.

### Identification of the tasiRNA3 target by RLM-5ʹ RACE

To identify *FaARF4* as a *tasiRNA3* target gene, 5ʹ-RNA ligase-mediated rapid amplification of cDNA ends (RLM-5ʹ RACE) was performed with the 5ʹ-Full RACE Kit (Takara, Dalian, China). According to the manufacturer’s instructions, PCR products were ligated into the pMD18-T vector, and the recombinant vectors were verified by sequencing. All primers used for RLM-5ʹ RACE are listed in Table S1.

### Rapid PCR site-directed mutagenesis of FaARF4

Rapid PCR site-directed mutagenesis was used to study *FaARF4* expression as previously described^28^. We mutated *FaARF4* with two pairs of mutant primers: FaARF4mut-F1/R1 and FaARF4mut-F2/R2 (Table S1).

### Transformation of *A. thaliana* and strawberry

The full-length *FaARF4*-coding region with added *Nde* I and *Sal* I restriction sites was amplified by PCR and inserted into the polylinker site of the plant overexpression vector pRI 101-AN. An RNAi vector, pART27, was constructed as previously described^45^. Briefly, a 333 bp fragment of *FveARF4* was inserted into the left (*EcoR* I and *Xho* I) and right (*Hind* III and *Xba* I) multiple cloning sites of the pART27 vector. All primers used are listed in Table S1.

*A. thaliana* was transformed using the *Agrobacterium tumefaciens* strain GV3101 and the floral dip method^20^. Transgenic plants were selected on 1/2 Murashige and Skoog (MS) medium with 30 mg/L kanamycin. Transgenic *A. thaliana* plants were grown in climate-controlled boxes at 24 °C under a 12/12 h light/dark cycle. *A. tumefaciens* strain EHA105 was used for ‘Ruegen’ transformation following the leaf-disk procedure^45^. Transgenic strawberry plants were planted in November in a greenhouse under sunlight, and the day and night temperatures were 15–25 °C and 5–10 °C, respectively.

### Identification of transgenic *A. thaliana* and strawberry

To identify transgenic *A. thaliana* and strawberry plants, genomic DNA was extracted using the CTAB method^44^. Transgenic plants were confirmed using the 35S-F/FaARF4-R primer set, targeting the pRI 101-AN vector and the transgene sequence. The following PCR conditions were used: 95 °C for 5 min, followed by 35 cycles of 95 °C for 30 s, 55 °C for 30 s, and 72 °C for 3 min, with a final extension at 72 °C for an additional 5 min. The expression of *ARF4* in the leaves of transgenic and control plants was analyzed using qRT-PCR with the *FaARF4* and *Fve26S*/*AtACTIN8* primers (Table S1).

### Yeast one-hybrid assay

The *FaARF4* sequence was ligated into the pGAD424 vector. The promoter sequences (1600 base pairs) of *FveAP1* and *FveFUL* were cloned and analyzed to determine whether AuxREs were present. Four *FveAP1* promoter fragments and four *FveFUL* promoter fragments were then amplified and individually inserted into the pAbAi vector. Primers are listed in Table S1. The vectors containing *FaARF4* and a fragment of the *FveAP1* or *FveFUL* promoter were cotransformed into the Y1H yeast strain, and the Y1H assay was performed as previously described^50^.

### Luciferase reporter assay

The *35S*::*FaARF4* and *35S*::*FveARF4* vectors were used as effectors, and the *FveAP1* and *FveFUL* promoters were inserted into the pRI-mini35S-LUC vector and used as reporters. Primers are listed in Table S1. The luciferase reporter assay was carried out following previously described methods^49^. Finally, the luciferase fluorescence and luciferase signal intensity were imaged and measured using a living fluorescence imager (Lb985, Berthold, Germany).

### Statistical analysis

The significance of the differences between the means was analyzed using Duncan’s test with DPS 7.05 software^48^. The mean values marked with * and ** indicate significant differences at the 5% and 1% levels, respectively.

## Supplementary information

Fig. S5

Table S1

Fig. S1

Fig. S2

Fig. S3

Fig. S4
