# Supplementary figures and images for: Auxin-induced AUXIN RESPONSE FACTOR4 activates APETALA1 and FRUITFULL to promote flowering in woodland strawberry
Source: Hortic Res. 2021 May 1;8:115. doi: 10.1038/s41438-021-00550-x (PMC8087778; doi:10.1038/s41438-021-00550-x)

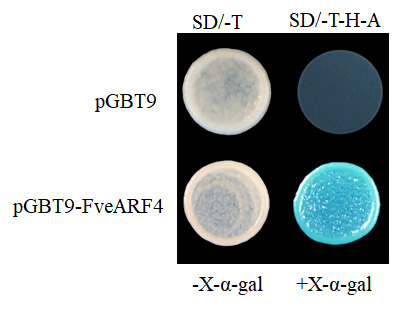

Supplement: Supplementary file 1 — Fig. S5 [file 41438_2021_550_MOESM1_ESM.jpg]

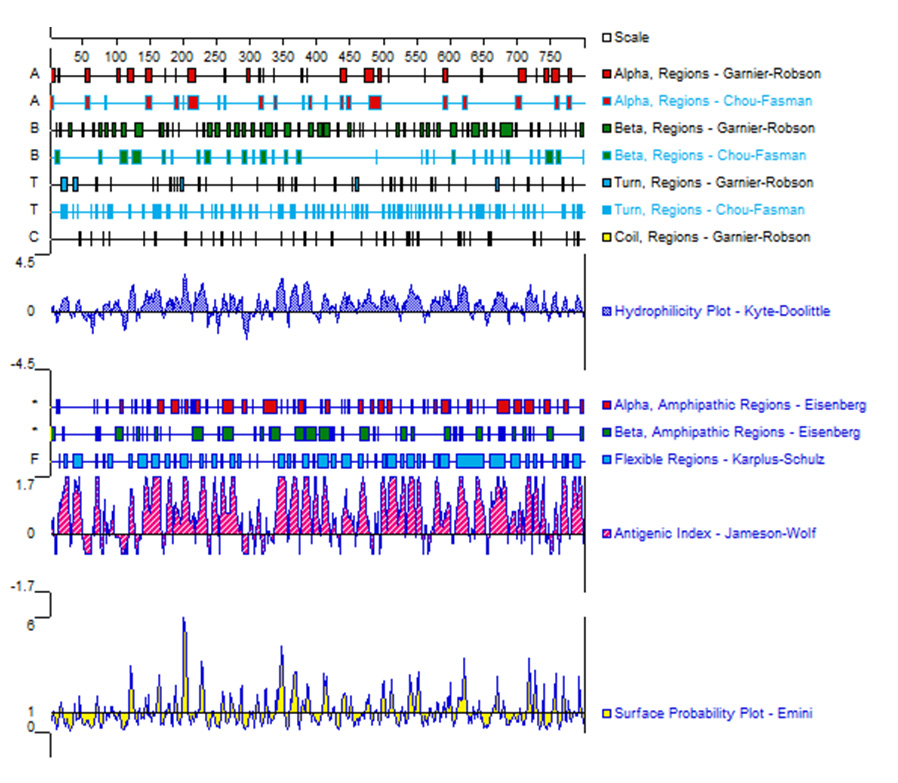

Supplement: Supplementary file 3 — Fig. S1 [file 41438_2021_550_MOESM3_ESM.jpg]

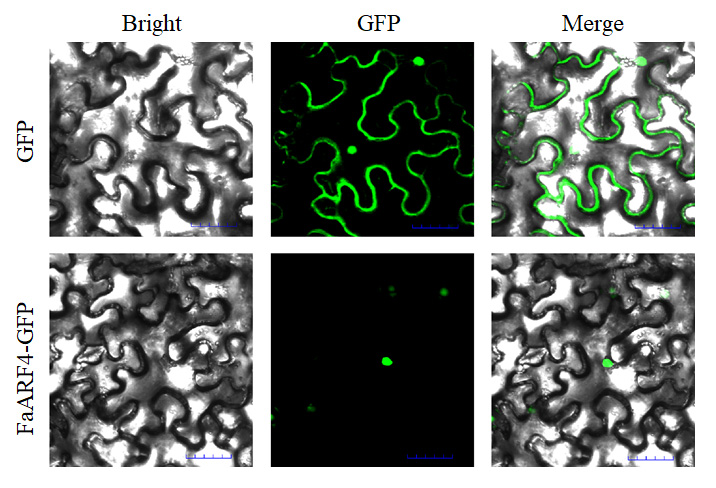

Supplement: Supplementary file 4 — Fig. S2 [file 41438_2021_550_MOESM4_ESM.jpg]

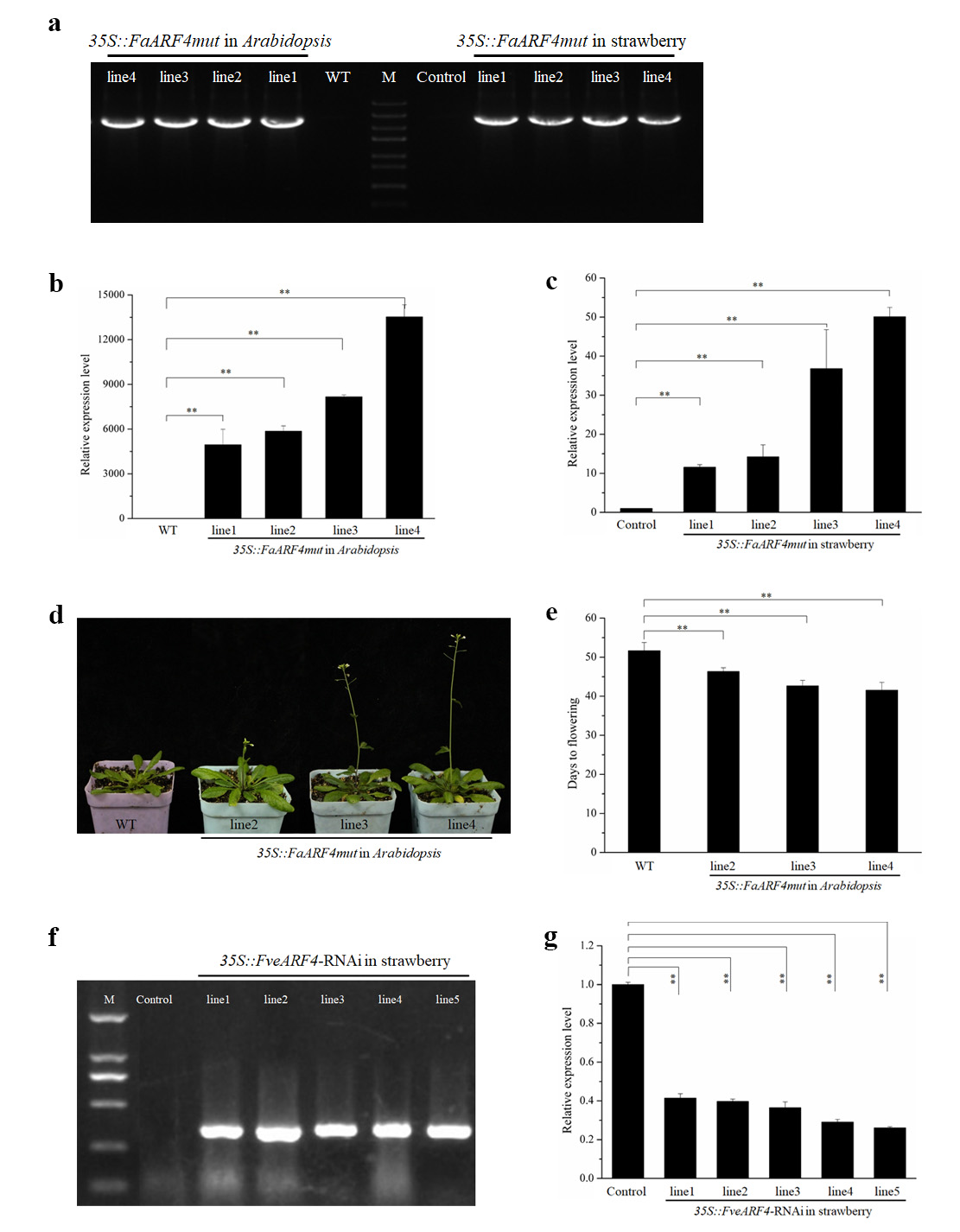

Supplement: Supplementary file 5 — Fig. S3 [file 41438_2021_550_MOESM5_ESM.jpg]

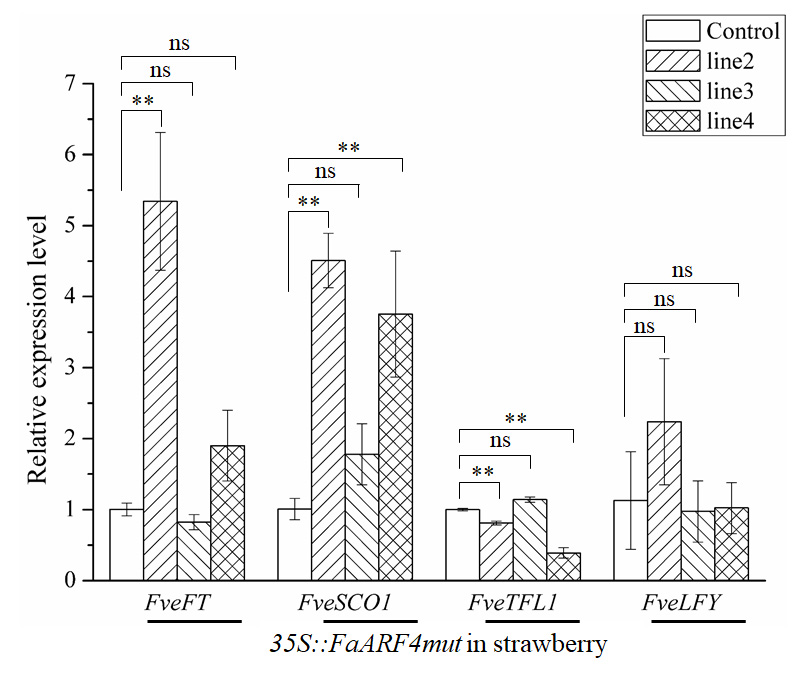

Supplement: Supplementary file 6 — Fig. S4 [file 41438_2021_550_MOESM6_ESM.jpg]
