# Supplementary material for: Auxin-induced AUXIN RESPONSE FACTOR4 activates APETALA1 and FRUITFULL to promote flowering in woodland strawberry
Source: Hortic Res. 2021 May 1;8:115. doi: 10.1038/s41438-021-00550-x (PMC8087778; doi:10.1038/s41438-021-00550-x)
Supplement: Supplementary file 2 — Table S1 [file 41438_2021_550_MOESM2_ESM.doc]

**Table S1** Primers and probes used in this study.

| Primer | Sequence (5’→3’) | Use |
| --- | --- | --- |
| FaARF4-F FaARF4-R | CGCCATATGATGGAATTTGATCTGA  GCGTCGACTCAGACCCTAATTGCTG | Clone of *FaARF4* CDS |
| FaARF3-QF  FaARF3-QR | GTCGTCGTCCAATTCGTCTG  TCATCACTACCACTCTCCGC | Real-time PCR of *FaARF3* |
| FaARF4-QF  FaARF4-QR | GACTGAAGAAACTGCGGACAAG  ttcttgaccttgcaagacc | Real-time PCR of *FaARF4* |
| Fa26S-F  Fa26S-R | TAACCGCATCAGGTCTCCAA CTCGAGCAGTTCTCCGACAG | Real-time PCR of *Fa26S* |
| AtACTIN8-F  AtACTIN8-R | TGCAGACCGTATGAGCAAAG  CTGGAAAGTGCTGAGGGAAG | Real-time PCR of *AtACTIN8* |
| Fve26S-F  Fve26S-R | TAACCGCATCAGGTCTCCAA CTCGAGCAGTTCTCCGACAG | Real-time PCR of *Fve26S* |
| FveFT-QF  FveFT-QR | TCGATCCTGATGCACCCA  TCCCAACTGCCGAAACAA | Real-time PCR of *FveFT* |
| FveSOC1-QF  FveSOC1-QR | GCAAGGAAGGCACAGGTT  CTGGCAGTCCAATGAACAAT | Real-time PCR of *FveSOC1* |
| FveTFL1-QF  FveTFL1-QR | TGTTCCTGGCCCTAGTGATC  AGGTTCGGGTGTTGAAGTGA | Real-time PCR of *FveTFL1* |
| FveAP1-QF  FveAP1-QR | CTCTGTCATGTGCGATGCTC  TCACCTTCAGCCTAGCATGT | Real-time PCR of *FveAP1* |
| FveFUL-QF  FveFUL-QR | TCAACGCTCCAGAAGAAGGA  CTCCCATTGTGACTGAGCCT | Real-time PCR of *FveFUL* |
| FveLFY-QF  FveLFY-QR | GAGGTGGTGGGCATAGAA  CTGATGGCACTGCTCGTAG | Real-time PCR of *FveLFY* |
| FaARF4-GFP-F  FaARF4-GFP-R | GCTCTAGAATGGAATTTGATCTGAACC  CCGCTCGAGGACCCTAATTGCTGTT | Sub-cellular localization of *FaARF4* |
| FaARF4-BD-F  FaARF4-BD-R | TGTATCGCCGGAATTCATGGAATTTGATCTGAACC  GCAGGTCGACGGATCCTCAGACCCTAATTGCTGTTG | Transcriptional activation analysis of *FaARF4* |
| FveARF4-BD-F  FveARF4-BD-R | TGTATCGCCGGAATTCATGGAATTTGATCTGAACC  GCAGGTCGACGGATCCTCAGACCCTAATTGCTGTTG | Transcriptional activation analysis of *FveARF4* |
| FaARF4-Inner1  FaARF4-Outer1  FaARF4-Inner2 FaARF4-Outer2 | CCAATAGTAGCCTTCTGCG  GTGAGCTCTCATGAACTCAC  CCAACCTTGTGAATGTCACC  GAGCACGTCATGGGATCTTG | RLM-5' RACE of *FaARF4* |
| FaARF4mut-F1  FaARF4mut-R1  FaARF4mut-F2  FaARF4mut-R2 | GACAGTTAAGTCCTCTAAAGTGTTACAGGGCCAGGAGAATATGGGTTTCATATCACCCCAC  GAAACCCATATTCTCCTGGCCCTGTAACACTTTAGAGGACTTAACTGTCTCCTCATAGTCC  GATAGATTTCCGAAGGTAAGCCAGGGCCAGGAGATATGCCCATTGAGATCCTTGTCAGG  CAATGGGCATATCTCCTGGCCCTGGCTTACCTTCGGAAATCTATCAGATTCCGCAAATCC | Site-directed mutagenesis of *tasiRNA3* target site in the *FaARF4* |
| FveARF4-RNAi-F1  FveARF4-RNAi-R1  FveARF4-RNAi-F2  FveARF4-RNAi-R2 | GGAATTCAGCTTCTGATACCAGTACCCATGGAGG  CCGCTCGAGTGGTTGCCAACAACTGAATCAGGAA  CCCAAGCTTTGGTTGCCAACAACTGAATCAGGAA GCTCTAGAAGCTTCTGATACCAGTACCCATGGAGG | Construction of *FveARF4* RNAi vector |
| 35S-F | GACGCACAATCCCACTATCC | Upstream primer of pRI101-AN |
| FaARF4-AD-F  FaARF4-AD-R | AAAAGAGATCGAATTCATGGAATTTGATCTGAACC  ATCTCTGCAGGTCGACTCAGACCCTAATTGCTGTTG | Y1H of FaARF4 |
| pFveAP1-F  pFveAP1-R  pFveAP1-1F  pFveAP1-1R  pFveAP1-2F  pFveAP1-2R  pFveAP1-3F  pFveAP1-3R | CGGGGTACCATTTAATCACAGCTAC  CCGCTCGAGAGGGGAATTTAGGATT  CGGGGTACCCACAGCTACAATCGTG  CCGCTCGAGGTCTCATAGTACAGGAG  CGGGGTACCGGGTTACCATACATGG  CCGCTCGAGCGTGAGTCGAACTCAT  CGGGGTACCTGAGTTCGACTCACGAAG  CCGCTCGAGGTTCAATACCACCGAATG | Fragment cloning of *FveAP1* promoter |
| pFveFUL-F  pFveFUL-R  pFveFUL-1F  pFveFUL-1R  pFveFUL-2F  pFveFUL-2R  pFveFUL-3F  pFveFUL-3R | CGGGGTACCATCCACGAAAGTATCCC  CCGCTCGAGTGTAAATTTATAGGAAAG  CGGGGTACCTTGAGCAAACGATTGG  CCGCTCGAGGCAATTCTCTTCGGTC  CGGGGTACCGCAACGCAGACAACAC  CCGCTCGAGGTGGAGTATCATGCTG  CGGGGTACCTTGGCCAAGAACACAC  CCGCTCGAGCCTCTGTGGGATTAGA | Fragment cloning of *FveFUL* promoter |
| pFveAP1-LUC-F  pFveAP1-LUC-R | CCCAAGCTTATTTAATCACAGCTAC  CGGGGTACCAGGGGAATTTAGGATT | Clone of *FveAP1* promoter |
| pFveFUL-LUC-F  pFveFUL-LUC-R | CCCAAGCTTATCCACGAAAGTATCCC  CGGGGTACCTGTAAATTTATAGGAAAG | Clone of *FveFUL* promoter |
